# A Scoping Review on Cucumis melo and Its Anti-Cancer Properties

**DOI:** 10.21315/mjms2024.31.4.5

**Published:** 2024-08-27

**Authors:** Raja Siti Syazana Raja Soh, Hermizi Hapidin, Mohamad Zahid Kasiram

**Affiliations:** 1 Department of Biomedical Science, Kulliyah of Allied Health Sciences, International Islamic University Malaysia (IIUM), Pahang, Malaysia; 2 Biomedicine Programme, School of Health Sciences, Universiti Sains Malaysia, Kelantan, Malaysia

**Keywords:** anti-cancer, Cucumis melo, half maximal inhibitory concentration (IC_50_), in vitro, in vivo

## Abstract

*Cucumis melo* L., better known by its popular cultivar cantaloupe, is an economically significant crop in the Cucurbitaceae family. Melon peel and seeds have shown medicinal potential due to their numerous biological qualities, including anti-inflammatory, anti-cancer, antibacterial, hepatoprotective and immunomodulatory effects to treat cardiovascular disease, diabetes and oedema. This scoping review aims to broaden the research scope on the cancer-fighting abilities of melon extract and its half maximal inhibitory concentration (IC_50_). Three databases which are Scopus, ScienceDirect and PubMed were used to locate relevant publications utilising the keywords ‘melon’, ‘*Cucumis melo*’, ‘inhibitory activity’, ‘cancer’ and ‘anti-cancer’. The Preferred Reporting Items for Systematic and Meta-analyses extension for Scoping Review (PRISMA-ScR) framework was used in conducting this study. Out of 904 articles, 14 articles met the inclusion criteria and were used in this analysis. These articles were published in English between 2000 and 2023 with full text accessibility, specifically addressed the fruit cantaloupe (*Cucumis melo* L.) or melon and reported on any type of cancer. *Cucumis melo* extract showed promising anti-cancer action in both in vitro and in vivo investigations on eight different cancer types: cervical, colon, prostate, leukaemia, multiple myeloma, breast, hepatoma and ovarian cancer. A thorough analysis shows that some of the IC_50_ values were significantly low, especially in cases of colon and prostate cancer, indicating a significant anti-cancer effect. The substantial anti-cancer benefits of *Cucumis melo* fruit extracts point to the necessity for additional investigation into their potential for cancer therapy on each form of cancer.

## Introduction

*Cucumis melo* L., commonly referred to as the well-known melon cultivar cantaloupe, is a commercially important crop belonging to the Cucurbitaceae family (1–3). The fruit parts are eaten, while the peels and seeds are discarded. According Rolim et al. (4), increasing fruit consumption increases the volume of waste generated, notably the peels and seeds. These melon peels and seeds have shown medicinal potential (4–6). For example, the oil extracted from seed contains a high concentration of vitamin E and polyunsaturated fatty acids, primarily linoleic acid (5). Melon seeds are also an excellent source of natural antioxidants and may serve as nutritional components or as a fortifying material to extend shelf life (6). For instance, melon seed flour (MSF) was shown to be a promising by-product with high nutritional value by Çağındı et al. (7). It can be used as a good source of protein, fat and fibre for the creation of fortified functional foods. These findings demonstrate that MSF has a low moisture content, which lowers spoiling and preserves microbiological quality while also aiding in the preservation of nutrients (8). It has been discovered that all components of the melon, even the seeds and peels, have their own health benefits.

Melon varieties have shown several biological properties towards human health. For many years, numerous traditional medicine systems have employed various melon species including *Cucumis melo* L. to cure a variety of illnesses, such as cardiovascular disease, diabetes and oedema. The melon also possesses anti-inflammation, anti-cancer, antibacterial, hepatoprotective and immunomodulatory properties (9, 10). Despite this, few studies have highlighted the anti-cancer properties of *Cucumis melo* for each form of cancer. The cytotoxicity of muskmelon fruit and seed extract has been studied (4, 11) though these studies were only conducted on one cancer cell type and extract from one plant part either seeds, peel or whole fruit (12). Therefore, a thorough scoping study of *Cucumis melo* and its anti-cancer properties was performed by reviewing and compiling the current literature on the anti-cancer effects of melon extracts and the half-maximal inhibitory concentration (IC_50_) values of these extracts.

## Methods

**Figure 1:**
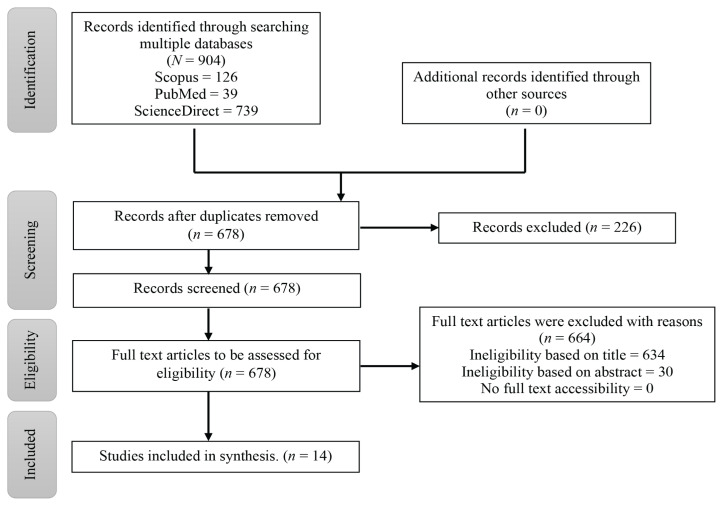
PRISMA-ScR by Tricco et al. (13) flow diagram of study selection

The purpose of this study was to provide a broad review of the most recent research on *Cucumis melo* extract and its anti-cancer properties. This scoping review was carried out in compliance with the Preferred Reporting Items for Systematic and Meta-analyses extension for Scoping Review (PRISMA-ScR) by Tricco et al. (13) which comprise identification, screening and eligibility, and included articles in the flow diagram (Figure 1).

### Identifying the Research Question

The review questions are: i) What are the anti-cancer properties of the melon? ii) What are the half-maximal inhibitory concentrations (IC_50_) values of melon extracts in the treatment of cancer cells?

### Distinguishing Relevant Studies

English academic journal articles published between 2000 and 2023 were searched using electronic database searches with PubMed, Scopus and ScienceDirect. The search included all study types but did not include systematic reviews or review articles. The researchers separately assessed the eligibility of titles, abstracts and keywords. Out of 904 articles found using electronic databases, 14 studies were included in this review. Figure 1 shows the flow diagram of data selection. The keywords used were ‘melon’ AND ‘inhibitory activity’, ‘melon’ AND ‘anti-cancer’, ‘melon’ AND ‘inhibitory activity’ AND ‘cancer’, and ‘*Cucumis melo*’ AND (‘anti-cancer’ OR ‘MTT assay’ OR ‘cell viability’) with Boolean terms AND/OR to combine or separate the keywords as shown in Table 1 and Table 2.

### Selecting Studies

The identified studies must satisfy the following eligibility criteria to be included in this review: i) fruits involved were of cantaloupe, *Cucumis melo* L. or melon; ii) reported on any type of cancer and iii) has full text accessibility.

### Charting the Data

The data were summarised in a table form which includes the author, year of publication, country, fruit parts, pure compound/crude extract, human cancer line/tumour xenograft, IC_50_, concentration used and main findings.

### Collating, Summarising and Reporting the Results

The collected results were subsequently compiled and summarised. Based on the study’s limitations that were discovered in the chosen articles, the research gaps were emphasised.

## Results

Three databases yielded a total of 904 publications: 739 publications in ScienceDirect, 126 publications in Scopus and 39 publications in PubMed (Figure 1). The articles were uploaded to Mendeley and checked for duplication. A total of 226 articles were removed and the remaining 678 articles were evaluated for eligibility based on the title and abstract. A total of 634 articles were excluded due to ineligibility determined by their titles and an additional 30 articles were excluded based on the ineligibility criteria outlined in their abstracts. Those articles were disregarded since they did not underline the anti-cancer properties or focus on *Cucumis melo*. Only 14 articles were included and further analysed because they matched the predetermined inclusion requirements.

### Characteristics of Study

From the year 2000 to 2010, only two studies (*n* = 2, 14.2%) were conducted regarding *Cucumis melo* and its anti-cancer properties. Since then, from 2011 to 2023, there have been a considerable rise in research (*n* = 12, 85.7%) evaluating the effectiveness of *Cucumis melo* extract as an anti-cancer agent (Table 3). China has the most discoveries in this field of study (*n* = 7, 50.0%) followed by Egypt (*n* = 2, 14.2%), Brazil, Iran, India, Japan and Taiwan (*n* = 1, 7.1%). All studies conducted in vitro evaluation (*n* = 14, 100%) including both human and animal cell lines and four of these also incorporated in vivo studies (*n* = 4, 28.6%)

A variety of human cancer cell lines were employed in an extensive number of in vitro research evaluations. The most used cancer cell lines were cervical cancer cells (HeLa), adenocarcinomic human alveolar basal epithelial cells (A549), (*n* = 3, 21.4%), followed by breast cancer cells (MCF-7), colorectal adenocarcinoma cells (HT-29), colon adenocarcinoma cells (HCT-116), prostate cancer cells (PC-3) (*n* = 2, 14.2%) and hepatoma cancer cells (BEL-7402), human non-small cell lung carcinoma (H1299), human lung fibroblast cells (HLF), human glioblastoma multiforme cells (GBM8401), colorectal carcinoma cells (RCM-1), lymphoblast cells (K562), cervical carcinoma cells (SiHa), kidney carcinoma cells (786-0), mouse lymphoma cells (L5178Y) and rat brain cells (PC12) (*n* = 1, 7.1%) (Table 4). Meanwhile, the in vivo assessments incorporated tumour xenograft in mice.

A wide range of assays were employed to assess the effectiveness of *Cucumis melo* extract on various cancer cell lines. 3-(4,5-dimethylthiazol-2-yl)-2,5-diphenyltetrazolium bromide assay (MTT) (*n* = 8, 57.1%) was the most used assay in the in vitro research followed by the Cell Counting Kit-8 assay (CCK-8), neutral red dye (*n* = 2, 14.2%) and 3-(4,5-dimethylthiazol-2-yl) -5-(3-carboxymethoxyphenyl)-2-(4-sulfophenyl)-2H-tetrazolium (MTS) assay, trypan blue exclusion assay, sulforhodamine B (SRB) assay (*n* = 1, 7.1%) (Table 5). Histological examination was done for in vivo studies. It comprised evaluation of tumour weight, assessment of angiogenesis of breast cancer in BALB/c mice using trypsin inhibitor from *Cucumis melo* seed extract and evaluation of morphology, which comprised evaluation of photomicrography (fluorescent staining method) and evaluation of histopathology (haematoxylin and eosin stain method).

### Assessment of Study Outcomes

All 14 articles reported positive findings on the anti-cancer properties of *Cucumis melo* extract on several cancer types (Table 6). Ten studies highlighted the anti-cancer properties of pure compounds such as cucumol A (14), cucumol B (15), cucurbitacin B (CuB) (16–19), protein trypsin inhibitor (20), cucurbitacin E (CuE) (21, 22) and (methylthio)acetic acid (MTA) (23) from the *Cucumis melo*. Seven studies highlighted the molecular pathways responsible for the cytotoxic and anti-proliferative effects of *Cucumis melo* extract on several cancer cell lines: triggering TLR4/NLRP3/GSDMD-dependent pyroptosis (inflammatory cell cytokines are released and cells inflate with bubbles as a form of programmed cell death) (19), arresting cell cycle at G_2_/M phase (18) via GADD45γ gene expression and blocking cyclin B1/CDC2 complex in GBM8401 (22), inhibiting expression of angiogenesis-related genes (20), reducing c-Raf (16), inducing STAT3-dependent apoptosis cell cycle (21) and triggering apoptosis (23).

In comparison to other cancer types, the anti-cancer activities of *Cucumis melo* extract against HCT-116 (12, 14) and HT-29 (4, 24) cell lines are the subject of the greatest research on melon anti-cancer properties (Table 7). In contrast to the study by Ibrahim et al. (14), which found an IC_50_ of 92.624 mg/mL for HCT-116, Zhang et al. (12) found the most potent IC_50_ at 0.25 mg/mL for seed chloroform extract and 0.26 mg/mL for whole fruit methanol extract (Table 8). Strong cytotoxic effects of *Cucumis melo* fruit extracts have also been reported by Ittiyavirah et al. (11) and Zhang et al. (12) against the PC-3 cell line. Ittiyavirah et al. (11) reported an IC_50_ of 1.47 mg/mL, whereas Zhang et al. (12) achieved an even more remarkable IC_50_ of about 0.34 mg/mL. These results demonstrate the noteworthy influence of *Cucumis melo* fruit extracts on prostate cancer and point to a promising direction for future studies in cancer therapy.

## Discussion

Research findings indicate that phytochemicals offer remarkable health advantages and are crucial in preventing human illnesses (12). The entire Cucurbitaceae family has several health benefits, with each fruit or vegetable having a distinct impact on human health (25). *Cucumis melo* is widely recognised for its advantageous pharmacological properties, which include anti-inflammatory, antioxidant, hepatoprotective, diuretic, anti-cancer, anti-ulcer and immunomodulatory effects (26). Notably, *Cucumis melo* contains bioactive substances that have shown anti-cancer effects, including cucumol A, cucumol B, phenolic compounds, protein trypsin inhibitor, MTA, CuB and CuE.

CuB is the most common and active compound among the cucurbitacin class and has attracted much interest from researchers worldwide (27). According to Zhang et al. (28) and Garg et al. (29), CuB activates the JAK/STAT, NF-κB, PI3K/AKT, Wnt/β-catenin and MAPK/ERK signalling pathways, causing apoptosis in a variety of cancer types. Particularly, the anti-cancer activity of CuB surpasses that of CuE, another well-studied component of cucurbitacin, as demonstrated by Jing et al. (21) and shows potential as a therapy agent for non-small cell lung cancer (30, 31). Further investigation by Yuan et al. (19) showed that CuB induced TLR4/NLRP3/GSDMD-dependent pyroptosis to suppress non-small cell lung cancer by elevating reactive oxygen species (ROS) and Ca^2+^, which may represent a viable target for treatment for this cancer type.

Additionally, Bajcsik et al. (32) identified CuE as a notable phytomolecule that is frequently present in therapeutic food plants from the Cucurbitaceae family. CuE is well known for its strong therapeutic potential. It has anti-inflammatory, immunomodulatory, hepatoprotective, and anti-cancer properties. For instance, an in vitro study by Hsu et al. (33) revealed that CuE therapy prevented brain metastasis of non-small cell lung cancer in mice model experiments, as well as the expression of yes-associated protein (YAP) and its downstream signalling genes in non-small cell lung cancer. CuE also had an anti-proliferative effect on A549 cells by acting as a tyrosine kinase inhibitor and interfering with the EGFR/MAPK signalling pathway (21).

This comprehensive scoping study evaluated the cytotoxic effects of *Cucumis melo* extract and its IC_50_ on several different cancer types. *Cucumis melo* might be capable of inhibiting nine different cancer types. The IC_50_ values of the extracts on the different cancer types ranged widely from 0.247 mg/mL to 178.384 mg/mL. Notably, the extract showed an outstanding IC_50_ of 0.24693 mg/mL on HCT 116 cell lines, indicating its potent inhibitory effect. The extract had the most inhibitory potency on HCT 116 when compared to the other cell lines (PC-3, Jurkat and HeLa) in the same investigation carried out by Zhang et al. (12). Furthermore, Li et al. (24) highlighted the correlation between stronger anti-proliferative efficiency and lower IC_50_ values.

Remarkably, the chloroform seed extract showed the greatest cytotoxicity, indicating an enhanced concentration of seed-derived bioactive substances. This association between higher levels of bioactive chemicals and enhanced cytotoxicity is consistent with previous research highlighting the direct connection between the quantity of active metabolites in plant extracts and their biological activities (34–36). Cucumol A, which is isolated from the seeds, showed significant cytotoxic action against HeLa cells and murine lymphoma cells (L5178Y) in a study conducted by Ibrahim et al. (14). Additionally, cucumol B, a new triterpene benzoate from *Cucumis melo* seeds, also acted as a cytotoxic agent on SKOV-3, MCF-7 and HCT 116 cells, as demonstrated by Ibrahim et al. (15). Terpenoids, especially those from cucurbitaceous plants, have been shown to exhibit chemopreventive and chemotherapeutic effects against various cancer types (15, 37).

Previously established research has demonstrated the efficacy of protease inhibitors in mitigating the risk of cancer development by inhibiting angiogenesis (38). A recent in vivo evaluation by Rezaei et al. (20) further substantiates this understanding, particularly focusing on a trypsin inhibitor extracted from *Cucumis melo* seeds. They showed that the trypsin inhibitor induced necrosis in tumour tissue, simultaneously suppressing the expression of pivotal genes associated with angiogenesis, such as matrix metalloproteinase genes (MMP-2, MMP-9) and vascular endothelial growth factor (VEGF). Moreover, the inhibitor manifested a dose-dependent positive influence on various tumour parameters, including height, width, depth and other crucial tissue characteristics.

This extensive body of research highlights the potential of *Cucumis melo* seed extracts as a source of various bioactive compounds with remarkable cytotoxic effects on a variety of cancer cell lines. These findings highlight the potential therapeutic use of trypsin inhibitors extracted from *Cucumis melo* seeds in treating cancer and provide insight into the complex ways in which these agents affect angiogenesis inhibition and other important aspects of tumour progression. The potentials of *Cucumis melo* extract as a powerful source of anti-cancer drugs are strengthened by this review. The data presented in this scoping review underscore the necessity of further investigation into the potential therapeutic uses of *Cucumis melo* in cancer treatment.

Even though most of the studies showed a positive effect of *Cucumis melo* on cancer treatment, this scoping review found a few research gaps. Firstly, the anti-cancer properties of melon extract have not been studied with respect to all types of cancer. This limitation in the scope of research raises the possibility that the observed treatment efficacy may be much more significant in malignancies that have not yet been investigated. Secondly, the publications did not emphasise the phytochemicals’ or the bioactive compound’s synergistic effects on prevention and treatment inside the crude extract. Thirdly, the evaluation of melon extract’s efficacy in treating cancer in vivo was limited to tumour xenografts. Prior to clinical trials, more information on in vivo study on dosages, administration modalities and immune system–cancer cell interactions is required. Finally, no clinical trial evaluations were provided despite the potent anti-cancer characteristics of melon extract in vitro and in vivo, highlighting the necessity for further investigation into the efficacy, efficiency, long-term impacts and safety prior to approving melon extract as a cancer treatment agent.

## Conclusion

This scoping study highlights the remarkable anti-cancer activities of *Cucumis melo* extract against a range of malignancies, including leukaemia, multiple myeloma, breast, hepatoma, ovarian, colon, prostate and cervical cancer. The measured IC_50_ values, which ranged from 0.247 mg/mL to 178.384 mg/mL, show the difference in the potency of *Cucumis melo* extracts against different cancer cells. Additionally, some bioactive compounds found in *Cucumis melo*, including cucumol A, cucumol B, CuB, CuE, protein trypsin inhibitor and MTA, significantly suppress the growth of certain cancer kinds. Notably, the evaluation primarily employed human cancer cell lines for in vitro studies, while murine models were utilised for in vivo investigations.

The scoping review reveals several remarkable IC_50_ values of *Cucumis melo* extract, particularly against colon and prostate cancer cells, suggesting a prominent anti-cancer effect. A comparison of the IC_50_ values from two distinct investigations revealed a pattern, with the most recent studies providing a more potent IC_50_ inhibitor value in contrast to the earlier research. For a thorough understanding, further combination studies are necessary to determine whether the in vitro synergy is also present in animal models. It is crucial to investigate drug interactions, especially in cases of prostate and colon cancers as it shows potential for future cancer therapy. Further research aimed at bridging the gap between in vitro and in vivo settings and exploring pathways in various cancer types will help us better grasp the therapeutic potential of *Cucumis melo* and explore the safety issues.

## Figures and Tables

**Table 1:** Keywords and search strings used in electronic database search

Keywords	Search strings
#1 melo	(*Cucumis melo*) OR (melon)
#2 anti-cancer	(anti-cancer) OR (cancer) OR (inhibitory activity) OR (MTT assay) OR (cell viability)
#3	#1 AND #2

**Table 2:** The process of selecting keywords in electronic databases through the utilisation of Boolean search operators

Keywords	Scopus	PubMed	ScienceDirect	Total
“melon” AND “inhibitory activity”	42	7	285	334
“melon” AND “anti-cancer”	55	25	269	349
“melon” AND “inhibitory activity” AND “cancer”	2	0	100	102
“*Cucumis melo*” AND (“anti-cancer” OR “MTT assay” OR “cell viability”)	27	7	85	119

**Total**				904

**Table 3:** Summary of the included studies (*n* = 14)

Characteristics	Number of studies
Year of publication	
January 2000 to December 2010	2 (14.1%)
January 2011 to September 2023	12 (85.7%)
Study location	
China	7 (50.0%)
Egypt	2 (14.1%)
Brazil, Iran, India, Japan, Taiwan	1 (7.1%)
Type of experiments	
in vitro	14 (100%)
in vivo	4 (28.6%)

**Table 4:** Type of cell lines and animals

Type of cell lines/animals	Number of studies
in vitro	
HeLa, A549	3 (21.4%)
MCF-7, HT-29, HCT-116, PC-3	2 (14.2%)
Jurkat, HepG2, SKOV-3, MM1.S, MM1.R, U266, RPMI 8266, BEL-7402, H1299, HLF, GBM 8401, RCM-1, K562, SiHa, 786-0, mouse lymphoma (L5178Y), rat brain (PC12)	1 (7.1%)
in vivo	
MC4-L2 cells in BALB/c inbred mice breast tissue, MM1.S cell in CB17-SCID mice, BEL-7402 tumour xenograft implant into nude mice, LLC cells into nude mice.	4 (28.6%)

Notes: HeLa = cervical cancer cells; A549 = adenocarcinomic human alveolar basal epithelial cells; MCF-7 = breast cancer cell lines; HT-29 = colorectal adenocarcinoma; HCT 116 = colon adenocarcinoma; PC-3 = prostate cancer; Jurkat = human T lymphocytes cell; HepG2 = hepatoblastoma cell line; SKOV-3 = human ovarian cancer cell line; MM1.S = proliferation of dexamethasone-sensitive; MM1.R = proliferation of dexamethasone-resistant; U266 = B lymphocyte isolated from the peripheral blood of a 53-year-old, male patient with myeloma; RPMI 8226 = B lymphocyte that was isolated from the peripheral blood of a 61-year-old, male with plasmacytoma; BEL-7402 = hepatoma cancer; H1299 = human non-small cell lung carcinoma cell line; HLF = human lung fibroblasts cell; GBM 8401 = human glioblastoma multiforme cells; RCM-1 = colorectal carcinoma; K562 = lymphoblast cells; SiHa = Cervical carcinoma cell line; 786-0 = kidney carcinoma; L5178Y = mouse lymphoma; PC12 = rat brain; MC4-L2 = mouse breast cancer cell line; SCID = severe combined immunodeficiency; LLC = Lewis lung carcinoma

**Table 5:** Type of assays used to assess anti-cancer activity

Type of assays	Number of studies
in vitro	
MTT assay	8 (57.1%)
CCK-8 assay, neutral red dye	2 (14.2 %)
MTS assay, trypan blue exclusion assay, SRB assay	1 (7.1%)
in vivo	
Histopathological examination	4 (28.6%)

Notes: MTT = 3-(4,5-dimethylthiazol-2-yl)-2,5-diphenyltetrazolium bromide assay; CCK-8 = Cell Counting Kit-8 assay; MTS = [3-(4,5-dimethylthiazol-2-yl)-5-(3-carboxymethoxyphenyl)-2-(4-sulfophenyl)-2H-tetrazolium]; SRB = sulforhodamine B

**Table 6:** Anti-cancer properties of *Cucumis melo* and its half maximal inhibitory concentration (IC_50_) of reviewed studies (*n* = 14)

No.	Author, year	Country	Fruit parts	Pure compound/crude extract	Human cancer cell line/tumour xenograft	IC_50_	Concentration used	Main findings
1.	Zhang et al., 2020	Agricultural field in China	*Cucumis melo* Seed	Crude extract	PC-3 HCT 116 Jurkat HeLa	0.3198 mg/mL 0.24693 mg/mL 0.26113 mg/mL 0.33133 mg/mL *chloroform extract	50, 150, 250, 350, 450 μg/mL	Chloroform extract had more soluble metabolites (polyphenol contents - gallic acid, flavonoid), hence it had better anti-cancer action than other solvents
			Whole fruit		PC-3 HCT 116 Jurkat HeLa	0.34101 mg/mL* 0.26486 mg/mL** 0.25021 mg/mL* 0.25770 mg/mL* *chloroform extract **methanol extract		
2.	Li et al., 2013	Markets in Guangzhou, China	Muskmelon (yellow pulp) Peel	-	A549 MCF-7 HepG2 HT-29	*No record since the author only state the strongest anti-proliferative effects on 4 cancer line	20, 40, 60, 80, 100 mg/mL from 200 mg/mL stock solution	0.89 0.70 0.78 0.88 *Anti-proliferative activities, high value indicates strongest inhibitory effects towards cell line
			Pulp		A549 MCF-7 HepG2 HT-29			0.88 0.70 0.96 0.86
			Seed		A549 MCF-7 HepG2 HT-29			0.89 0.83 0.94 0.90
3.	Ibrahim et al., 2016	El-Galaa village, Samalout, Minia, Egypt.	*Cucumis melo* L. *var reticulates* Seed	Pure compound- Cucumol A	Mouse lymphoma (L5178Y) Rat brain (PC12) HeLa	-	50μL	Cucumol A showed cytotoxic activity towards L5178Y and HeLa cells with median effective dose (ED_50_) values of 1.30 and 5.40 μg/mL, respectively compared to paclitaxel (0.07 and 0.92 μg/mL), respectively
4.	Ibrahim et al., 2019	Mankabad, Assiut, Egypt.	*Cucumis melo*. L Seed	Cucumol B	SKOV-3 MCF-7 HCT-116	22.96 mg/mL 4.48 mg/mL 92.624 mg/mL (moderate)	-	The extracted triterpene from *C. melo*’s cytotoxic properties support its use as a cytotoxic agent against human breast and ovarian cancer
5.	Yang et al., 2017	Sigma Aldrich (Cucurbitacins)	*Cucumis melo* Stem/pedicle	Cucurbitacin B	MM1.S MM1.R U266 RPMI 8226 CB17-SCID mice inoculated with MM1.S cell	5.36 mg/mL 1.6 mg/mL 1.68 mg/mL 4.48 mg/mL	0, 25, 50, 100, 200 nM	CuB interferes with multiple cellular pathways in multiple myeloma (MM) cells In a mouse model of MM, CuB decreased the growth of MM tumours without causing host toxicity, suggesting that it could be a potential new therapeutic agent for the treatment of MM
6.	Chan, Meng, et al., 2010	Huisong Pharmaceutical	*Cucumis melo* Stem/pedicle Bought Cucurbitacin B	Cucurbitacin B	BEL-7402 cells BEL-7402 tumour into nude mice	178.384 mg/mL	0.01, 0.1, 1, 10, 100, 1000 μM	Cucurbitacin B can reduce c-Raf and slow cell proliferation without inhibiting STAT3 on BEL-7402 cells Oral administration of cucurbitacin B has also been shown to be effective. Results showed that oral administration of cucurbitacin B at 0.5 and 1 mg/kg significantly inhibited tumour growth in almost half of the treated mice
7.	Yuan et al., 2021	Chengdu, China	*Cucumis melo* Pedicle	Cucurbitacin B	A549 H1299 HLF LLC cells injected into mice		0, 10, 100, 1000 nM	CuB inhibits NSCLC in vivo and in vitro by triggering TLR4/NLRP3/GSDMD-dependent pyroptosis
8.	Chan et al., 2010			Cucurbitacin B was obtained from ChromaDex, Inc	K562 * was chosen for additional study since it had the lowest growth inhibition (GI_50_) value among the five cell lines.		1 μM to 50 μM	Cucurbitacin B suppresses the Raf/MEK/ERK pathway in human leukaemia cells in addition to the STAT3 pathway. This finding offers important insight into the molecular mechanism underlying cucurbitacin B’s anti-cancer activity and strongly suggests that cucurbitacin B is a promising drug candidate for the treatment of human leukaemia
9.	Rezaei et al., 2022		*Cucumis melo* Seeds	Protein trypsin inhibitor (TI) Extract seed powder (EXT)	MC4-L2 cells in BALB/c inbred mice breast tissue	TI - 0.3 mg/mL EXT-0.4 mg/mL	5, 10, 25, 50, 100, 200, 300, 400, 800, 1200 μg/mL for both (TI) and (EXT)	TI, EXT could inhibit the expression of angiogenesis-related genes such as matrix metalloproteinase gene, MMP-2, MMP-9, VEGF and increase tumour tissue necrosis Generated dose-dependent improvements in the tumour’s height, width, depth, and other tumour tissue parameters The biggest reduction in tumour size was observed with combination therapy using TI and tamoxifen (TAM), which also inhibited tissue necrosis and the expression of genes related to angiogenesis
10.	Jing et al., 2020	Shanghai, China	*Cucumis melo*. L	Cucurbitacin E	A549	*No cytotoxicity test done	0, 0.25, 1.0, 2.5 μmol L^−1^	CuE induced STAT3 dependent apoptosis cell cycle arrested at G_1_/G_0_ phase. (shown its anti-proliferative effect)
11.	Hsu et al., 2014	Sigma, USA	*Cucumis melo*	CuE	GBM 8401	–	0, 2.5, 5, 10 μM	CuE inhibits tumour growth by arresting cell cycle at G_2_/M phase via GADD45γ gene expression and blockade of cyclin B1/CDC2 complex in GBM8401 cells
12.	Kamimura et al., 2023	Kyoto, Japan	*Cucumis melo var. conomon* (Japanese pickling melon)	Methylthio acetic acid (MTA)	RCM-1	–		MTA triggers differentiation and apoptosis on RCM-1
13.	Rolim et al., 2018	-	*Cucumis melo* L. reticulatus Peel	Phenolic compounds	SiHa HeLa HT-29 786-0	1.2 mg/mL 0.5 mg/mL 4.0 mg/mL 0.4 mg/mL	0.1, 0.25, 0.5, 1.0 mg/mL	Melon’s peels and seeds have potential as promising anti-tumour agents
			Seed		SiHa HeLa HT-29 786-0	0.4 mg/mL 0.3 mg/mL 2.8 mg/mL 1.0 mg/mL		
14.	Ittiyavirah et al., 2014	Cherthala, Alappuzha	*Cucumis melo*. L. Fruit	Crude extract	PC-3	1.47 mg/mL	100, 500, 1000 μg from 100 mg/mL	On a metastatic human prostate cancer line (PC-3), an aqueous extract of *C. melo* was found to have dose-dependent cytotoxic action

Notes: Abbreviations used same as Table 4

**Table 7:** Studies of anti-cancer effects of melon on several types of cancer

Parts of melon/phytochemical	Types of cancer	Number of studies
Seed	Colorectal cancer/colon cancer	4
	Cervical cancer, breast cancer	3
	Prostate cancer, leukaemia, lung cancer, hepatoma, lymphoma, brain cancer, ovary cancer, kidney carcinoma	1
Fruit/pulp	Prostate cancer, colon cancer	2
	Leukaemia, cervical cancer, hepatoma, breast cancer	1
Peel	Lung cancer, breast cancer, hepatoma, colon cancer, cervical cancer, colorectal cancer, kidney carcinoma	1
Stem - CuB	Multiple myeloma, hepatoma, non-small cancer lung cancer, leukaemia	1
Others:		
•CuE	Lung cancer, brain malignant glioma	1
•MTA	Colorectal cancer	1

Notes: CuB = Cucurbitacin B; CuE = Cucurbitacin E; MTA = methylthio acetic acid

**Table 8:** Anti-cancer effects of melon on several type of cancer with its half maximal inhibitory concentration (IC_50_)

Types of cancer	IC_50_
Colon adenocarcinoma (HCT-116)	92.624 mg/mL 0.24693 mg/mL a 0.26486 mg/mL b
Colorectal adenocarcinoma (HT-29)	4.0 mg/mL 2.8 mg/mL
Prostate cancer (PC-3)	1.47 mg/mL 0.3198 mg/mL a 0.34101 mg/mL a
Cervical carcinoma (SiHa)	1.2 mg/mL 0.4 mg/mL
Cervical cancer (HeLa)	0.5 mg/mL 0.3 mg/mL 0.33133 mg/mL a 0.25770 mg/mL a
Breast cancer (MCF-7)	TI - 0.3 mg/mL c EXT-0.4 mg/mL c 4.48 mg/mL
Multiple myeloma	5.36 mg/mL 1.6 mg/mL 1.68 mg/mL 4.48 mg/mL
Hepatoma cancer (BEL-7402)	178.384 mg/mL
Ovarian cancer (SKOV-3)	22.96 mg/mL
Leukaemia (jurkat)	0.26113 mg/mL a 0.25021 mg/mL a
Kidney carcinoma (786-0)	0.4 mg/mL 1.0 mg/mL

Notes:

a chloroform extract,

b methanol extract,

c in vivo, MC4-L2 cells in BALB/c mice
